# The Application of Stem Cells from Different Tissues to Cartilage Repair

**DOI:** 10.1155/2017/2761678

**Published:** 2017-12-10

**Authors:** James N. Fisher, Irene Tessaro, Tommaso Bertocco, Giuseppe M. Peretti, Laura Mangiavini

**Affiliations:** ^1^IRCCS Istituto Ortopedico Galeazzi, 20161 Milan, Italy; ^2^Università Vita-Salute San Raffaele, Milan, Italy; ^3^Department of Biomedical Sciences for Health, University of Milan, Milan, Italy

## Abstract

The degeneration of articular cartilage represents an ongoing challenge at the clinical and basic level. Tissue engineering and regenerative medicine using stem/progenitor cells have emerged as valid alternatives to classical reparative techniques. This review offers a brief introduction and overview of the field, highlighting a number of tissue sources for stem/progenitor cell populations. Emphasis is given to recent developments in both clinical and basic sciences. The relative strengths and weaknesses of each tissue type are discussed.

## 1. Introduction

Articular cartilage has a poor self-healing potential, mainly due to the lack of vascularisation and the paucity of undifferentiated cells [1]. Thus, if focal cartilage lesions are left untreated, they can progress to more extensive defects and may ultimately require treatment with joint replacement surgery if conservative options fail. The aim of this review is to describe in detail recent findings in both basic and clinical studies that have adapted cells from a variety of cell sources to cartilage repair strategies.

Current treatments for cartilage repair are mainly focused on bone marrow stimulation techniques: such as abrasive chondroplasty, subchondral drilling, microfracture and, more recently, nanofractures [2]. The aim of these techniques is to allow migration to the damaged area and the subsequent chondrogenic differentiation of multipotent bone marrow-derived stromal/stem cells (BMSCs). However, often, the regenerated tissue does not possess the same biochemical and biomechanical properties of the native cartilage; therefore, it is not able to resist the continuous stresses placed upon it, and it quickly degenerates [3]. Hence, new treatment options for articular cartilage lesions have grown in recent decades, due to promising results obtained with the development of new therapeutic options.

Tissue engineering strategies aim to regenerate the damaged tissue and restore a biologically and biomechanically valid articular surface. This requires three components, which may be alternately combined. The first is a suitable cell source which can differentiate into, and maintain, the specific cell phenotype; research in this area forms the body of this review (see Table 1). Additionally, signalling molecules such as growth factors, cytokines, or hormones stimulate cell growth and differentiation, and traditionally, a scaffold is used to provide an adequate three-dimensional environment [4, 5], although scaffold-free techniques have also proven successful (reviewed in Shimomura et al. [6]).

Growth factors, cytokines, and hormones are used to stimulate cell proliferation (owing to the low number of endogenous progenitors) and induce chondrocytic differentiation (without inducing hypertrophy or causing transformation) leading to the secretion of a collagen-rich extracellular matrix (ECM). Growth factors exert their effects by binding to, and activating, specific membrane-bound (usually transmembrane) receptors. Ligand binding typically leads to the activation of an intracellular signalling cascade (such as MEK/ERK, protein kinase C, and PI3K/AKT) and/or transcription factors, resulting in altered gene expression. Genes related to proliferation and differentiation are common targets of fibroblast growth factor 2 (FGF-2), which has been utilized for BMSC expansion [7], while insulin-like growth factor 1 has been applied to chondrogenic differentiation of peripheral blood (PB) cells [8] and to the repair of cartilage defects in rabbits [9]. Members of the TGF-*β* superfamily, which include TGF-*β*1 and BMPs 2, 4, and 7, have been shown to influence the development of cartilage [10] but may skew differentiation towards hypertrophic chondrogenesis and endochondral ossification [11], and TGF-*β* has been linked to cancerous progression in humans [12]. Alternatively, growth differentiation factor 5 has been shown to regulate the differentiation of articular chondrocytes [11] at least in part through inhibition of the BMP4 pathway [13].

Methods for the isolation and preparation of stromal cell populations are not standardized. Indeed, the method of isolation and preparation and the degree of *ex vivo* manipulation vary widely between laboratories and donor tissue source. Generally, tissue samples are harvested in sterile conditions, and cells are isolated with by different methods (enzymatic digestion or direct culture). Subsequently, cells are cultured *in vitro* with different conditions. The most popular method to induce chondrogenesis consists in of pellet culture with conditioned medium, which is enriched with insulin, dexamethasone, ascorbic acid, sodium pyruvate, and growth factors, such as TGF-*β*s or BMPs [14]. Chondrogenesis is then confirmed by the analysis of the extracellular matrix (production of GAGs) and by gene expression of cartilaginous markers (i.e., collagen type II, Sox-9).

The choice of scaffold material is significant as the 3D microenvironment is important for the correct growth and differentiation of cells [5, 15–18]. This microenvironment includes not only the materials which constitute the scaffold and their characteristics (such as porosity [19], rigidity [18], and biodegradability [16]) but also the *in vitro* culture conditions (media formulations, as well as both hydrostatic and mechanical forces [20, 21] and oxygen levels [22] that cells are exposed to).

Thus, growth factors and scaffolds are often combined with cells for regenerative purposes. For cartilage repair, several cell sources are already available and others are rapidly emerging; the aim of this manuscript is to provide an overview of recent developments in the field, with a particular focus on stem cells.

## 2. Terminally Differentiated Cells or Multipotent Cells for Cartilage Repair

Articular chondrocytes have been extensively used in the past years for autologous chondrocyte transplantation. However, the use of articular chondrocytes is limited by several factors: morbidity at the harvest site, the requirement of a second surgical procedure, and cell dedifferentiation due to *in vitro* expansion [4, 23–25], necessitated by the limited number of harvestable cells. Alternative sources of differentiated chondrocytes have been investigated, and recently, in a first-in-human trial, autologous nasal septum chondrocytes were used for the repair of full-thickness articular cartilage defects of the knee [26]. At 2-year follow-up, the changes in a range of clinical scores (IKDC, KOOS pain, KOOS symptoms, KOOS function in daily living, KOOS sport, and KOOS quality of life, relative to preintervention) were positive and the safety of the procedure was confirmed [26]. A phase II clinical trial (NCT01605201) is currently underway to confirm these data.

Stem cells are a cell source of vast potential, which can be isolated from a range of different tissues. These cells constitute a self-renewing population, which can undergo multilineage differentiation [27]. Pluripotent embryonic stem cells derive from the fertilization of the egg, and they can differentiate into any of the three germ layers (endoderm, mesoderm, or ectoderm); thus, they possess the potential to differentiate into any cell lineage. The role of these cells for tissue engineering has been investigated since the late ‘90s; however, along with induced pluripotent stem cells (iPSCs), the potential tumourigenicity and ethical issues have limited their use in clinical practice (with the notable exception of umbilical cord-derived stem cells [28, 29]). Conversely, adult postnatal stem cells can be more easily utilized for tissue engineering. These cells have a limited self-renewal and multilineage potential [27, 30, 31], but they can be isolated from individuals of any age without the ethical dilemmas of embryonic stem cells. The term “mesenchymal stem cell' (MSC) [32] describes a specific subpopulation of adult stem cells on the basis of established “minimal criteria” identified by the International Society for Cellular Therapy (ISCT) [33, 34] including several cell-surface markers, adherence to plastic culture dishes, and the potential to differentiate into chondrogenic, osteogenic, myogenic, adipogenic, and tenogenic lineages. Cell populations conforming to these criteria can be isolated from several tissues: bone marrow, synovium, adipose tissue, periosteum, peripheral blood, and umbilical cord blood, as well as from the inner part of cartilage of the knee. It must, however, be pointed out that often, these cell populations which are labelled “stem cells” would be more accurately described as stem cell-containing populations. Frequently, the multipotency and self-renewal capacity of these cells are not reported despite the existence of simple tests to do so, such as the colony-forming assay (CFU). The number of actual stem cells isolated from tissue can vary enormously depending on the age of the patient, the technique used for isolation, and the source tissue [31]. The omission of this data makes it challenging to assess the true role of the stem cell as opposed to stromal cells in these studies and to make meaningful comparisons between different studies [35]. Finally, the term “MSC” is sometimes used with no additional information as to the tissue of origin, while stromal cell populations isolated from the bone marrow or adipose tissue, for example, may both conform to the ISCT criteria for “MSCs”; they differ at the epigenetic [36] and phenotypic levels [37, 38] making the inclusion of this information crucial.

The chondrogenic potential of numerous stem cells has been analysed with regard to their possible use in tissue engineering. Probably, the most obvious source of stem cells to regenerate cartilage tissue is cartilage itself, and many studies have sought to isolate and harness the regenerative power of cartilage-resident stem/progenitor cells, some with great success [39–41] (see Table 1). Early studies followed from the illustration of the multilineage potential of BMSCs [27] culminate at the end of the last century with the demonstration of the exclusive and stable differentiation of clonal BMSC populations into chondrocytes [41]. Since then, researchers have capitalised the diversity of tissues from which stem/progenitor cells can be extracted.

**Figure 1:**
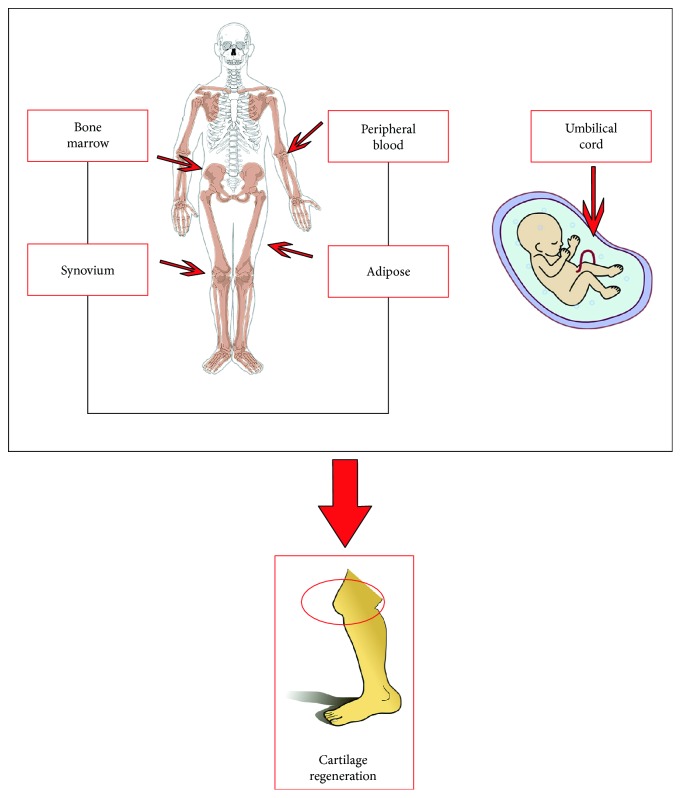
Stem cell sources for cartilage repair.

Hereafter, we will singularly describe the different tissue sources of stem cells (Figure 1).

### 2.1. Cartilage

Tissue engineering strategies utilizing autologous cartilage-derived stem/progenitor cells have been attempted since the late 1980s [42–45]. The largely acellular character of cartilage [44] combined with the scarcity of progenitors has been a hurdle to its use; however, some success has been seen using the cells resident in the articular cartilage of the knee [42, 45] and the jaw [39]. The advantage of chondrocytes and cartilage-resident cells is their ability to survive in the hypoxic environment found in the wound/implant. Successful results have also been reported using cells taken from the nasal septum [26, 46–48].

The articular cartilage of the knee is a thin layer of largely acellular connective tissue that protects and facilitates the movement of the joints [44]. Due to the low number of resident progenitor cells and challenges in defining the characteristics of the cartilage stem/progenitor cell [1], cartilage isolates have proven unconducive to *in vitro* cartilage production and any *in vitro* manipulation must be checked for unintended subsequent osteogenesis or tumourigenesis after implantation.

Recently, resident cartilage progenitor cells isolated from autologous cartilage tissue were shown to form tissue with the characteristics of hyaline cartilage when implanted ectopically in a mouse model; this was supported by data from high-density 2D cultures [39]. These cells were expanded *in vitro* and implanted in the knees of patients on a collagen scaffold. Patients reported significant improvements (using both IKDC and Lysholm scoring systems); importantly, MRI indicated that the implants covered the defect site and that no sign of hypertrophy was present; histological examination of a subset of implants showed no calcification, inflammation, or vascularisation. In addition to the improved clinical scores, 14 of 15 patients resumed sports activities within 1 year of the intervention, indicative of the practical value of this technique for improving patient quality of life.

Another study overcame the paucity of resident fibrocartilage stem cells (FCSC) within the jaw articular cartilage through prolonged *in vitro* culture [37]. Animal studies showed that a single FCSC was capable of generating a cartilage template that was remodelled into bone and a bone marrow space, including the haematopoietic microenvironment, without exogenous stimulation from osteogenic scaffolds (such as hydroxyapatite), Matrigel or factors, such as BMPs. This would seem to be great news for bone tissue engineers, but not so great for cartilage regeneration as formation of bone within the articulation is hardly an ideal outcome. However, the authors describe the mechanism by which the pool of resident FCSCs is maintained, though the inhibition of WNT signalling by sclerostin. Indeed, application of sclerostin favoured the differentiation of FCSCs into mature chondrocytes and aided cartilage repair in a rabbit model of cartilage injury [37].

The potential of cells from the nasal septum cartilage for tissue engineering applications was hinted at by basic studies from 2011 to 2012 [46, 48]. Through *in vitro* experiments and mouse studies, the inherent chondrogenic potential of nasal chondrocytes (NCs) was shown to be similar to that of matched BMSCs with NCs undergoing chondrogenesis in pellet culture without stimulation from either TGF-*β* or BMPs [46]. Significantly, NCs retained their chondrogenic abilities for far longer, until passage 35, in line with observations that NCs displayed lower levels of senescence markers than BMSCs [48] which would indicate that NCs could be advantageous for tissue engineering strategies that call for multiple rounds of ex vivo expansion. Dedifferentiated NCs have been shown to have greater clonogenic potential (over 3-fold more) and to proliferate faster that articular chondrocytes [38]. Unlike BMSCs, NCs were not susceptible to adipogenic induction [46, 48], possibly due to the significantly higher levels of BMP2 mRNA in NCs [48]. *In vivo*, NCs displayed no tumourigenicity or signs of metastasis in mice after 4 months, and clinical data show that autologous *ex vivo*-expanded NCs filled the defect and had no signs of delamination after a similar period of time [38].

### 2.2. Bone Marrow

Substantial clinical information is available on the suitability of bone marrow stromal cells (BMSCs) for cartilage tissue engineering. From initial results showing the potential for cartilage repair [41] to multiple clinical trials [45, 49–54], there is ample evidence to illustrate the applicability of BMSCs to cartilage defect repair. The majority of studies have focused on the use of autologous cells [45, 49–51, 53, 54]; however, there are also instances of successful application of allogeneic stem cell preparations to cartilage repair [52].

Removal of the bone marrow is usually achieved by aspiration from the iliac crest of the pelvis. While this is less invasive than some other methods of harvesting cells, the number of stem/progenitor cells obtained is not high and some form of expansion is often performed to obtain sufficient cell numbers. Aside from the concerns about the loss of cell multipotency during 2D cell culture [55], this remains the standard method for expansion of BM stromal cell samples. In some cases, the 2D expanded cell population is then embedded or seeded on a scaffold which provides rigidity and form, before being implanted at the defect site: the choice of scaffold material is not trivial and may influence the differentiation of the embedded cells [56]. The efficacy of 2D expansion followed by implantation on a cartilage-based scaffold has been demonstrated in the lab and in the clinic [45, 49, 51, 53, 54] with follow-up times up to 11 years [53]. Clinically scaffold-based BMSC implantation resulted in significant improvements in various indicators of quality of life and joint function, including increased mobility and reduction of pain. Although not all patients are willing to undergo second-look arthroscopy to assess cartilage formation and coverage, some data exists which shows that some defects were filled with fibrocartilage [49]. Scaffold-free administration of BMSCs expanded *in vitro* to form a “cell sheet” has also been shown to be effective in an animal model of cartilage defects at 12 weeks [7]. Here, FGF-2, in combination with chondrogenic factors, was noted to increase chondrogenic differentiation as well as cell growth [7]. Intra-articular (i.a.) injection of BMSCs alone [57, 60, 61] or with additional materials (such as hyaluronic acid (HA)) [59] has been applied to cartilage repair in clinical studies. The results were mostly positive, with an improvement in articular cartilage and meniscal repair noted in patients treated with BMSCs as opposed to controls (when analysed by IKDC, Tegner, and Lysholm scores, as well as MRI and MOCART scores in addition to evaluations of pain and quality of life) [50, 54].

Owing to the low frequency of stem/progenitor cells within the BM and the period of time required for *in vitro* expansion (typically several weeks), an alternate approach has been used for bone marrow aspirate concentrate (BMAC) [49, 50]. This technique has produced mixed results for the treatment of both osteoarthritis (OA) and cartilage defects in the knee. Gobbi and colleagues describe a case series with significant improvements in multiple scoring matrices (Tegner, Marx, Lysholm, VAS, IKDC subjective, and KOOS scores: *P* > 0.001) at 41 months postoperation (postop), relative to the same tests prior to the intervention [49]. On the other hand, Shapiro et al., in a randomised controlled trial for the treatment of bilateral OA, injected patients with either saline or BMAC, with follow-up at 6 months only to find that the level of pain relief afforded was similar in both treatment and control groups [50]. An additional technique involves the *in vitro* use of FGF-2 to rapidly expand autologous BMSCs to the point where it is feasible to generate a scaffold-free osteochondral implant thus partially overcoming the often limiting number of BMSCs obtainable from patients [7].

Caution must be exercised when using cells derived from the BM for cartilage repair, as the cells that generate hyaline cartilage are distinct from the growth plate chondrocytes found in the BM which form hypertrophic cartilage that is then remodelled into bone [13, 57–60]. Also, there is evidence suggesting that the differentiation and colony-forming potential of BMSCs decrease with donor age, a potential hurdle for autologous use in the elderly [61].

Future prospects for the use of BMSCs for cartilage engineering include the application of 3D printing technologies to tissue engineering with various groups reporting on the fabrication of 3D scaffold materials [62–64]. Recently, the concept has been taken to the next logical step, and a mixture of viable BMSCs and various polymers was used to create a 3D ECM containing live cells which survived *in vivo* and expressed markers of chondrocytic differentiation [62].

### 2.3. Synovium

It has been shown, via lineage tracing, that articular chondrocytes derive from synovial joint progenitors, or interzone cells [57], which do not contribute to the growth plate and thus to the formation of bone though endochondral ossification. This represents an advantage in the field of cartilage tissue engineering as heterotopic ossification is to be avoided. The development of articular chondrocytes, as opposed to hypertrophic chondrocytes, has been shown to be influenced by the TGF-*β* pathway, as opposed to signalling through BMP4 [13].

Synovial cells have been assessed for their use in cartilage repair, although few clinical data are published. Basic studies in animal models however are promising showing that synovium-derived cells represent a valid option for continuous study. Mak et al. found a population of synovial sca-1^+^ progenitor cells with inherent chondrogenic potential which were shown to increase cartilage repair 4 weeks after i.a. injection in a mouse model [65], while Baboolal and colleagues present results suggesting that HA present in the synovial fluid inhibits the initial interaction between stromal cells and cartilage [66]. These last results may be significant as inhibition of early binding events could be deleterious for the repairing potential of injected cells. Indeed, a series of studies from researchers at Tokyo Medical and Dental University in Japan have illustrated the significance of early cell attachment through the use of their “local adherent technique” whereby a short period (10 minutes) of joint immobility is sufficient for improved attachment of synovium-derived stem cell populations and results in significantly improved healing in both nonhuman animals and clinical studies [67–69].

### 2.4. Adipose Tissue

In 2001, Zuk et al. demonstrated that adipose-derived stromal/stem cells (ADSCs) can be differentiated into chondrocytes, adipocytes, and osteoblasts [70] paving the way for a host of studies into the application of autologous ADSCs in regenerative medicine [71–73]. As in the neighbouring field of bone regenerative medicine, where proponents of BMSC-based or ADSC-based cell therapies cite the merits of either tissue versus the other [40], the same is true for cartilage engineering. There are parallels between the fields, and the various merits are somewhat overlapping. On the one hand, the accessibility and abundance of adipose tissue are an obvious advantage over the limited volumes that can be collected from the bone marrow, with less comorbidity to boot. ADSCs were shown to have a higher clonogenic potential and lower tendency towards osteogenic differentiation [74]. On the other hand, the regenerative potential of ADSCs versus BMSCs, millilitre for millilitre, appears to favour the use of the less abundant, harder to access, BMSCs. Indeed, *in vitro* comparisons of the chondrogenic potential of human BMSCs and ADSCs have concluded that BMSCs possess a greater chondrogenic potential than matched ADSCs [75, 76]. An important paper from 2010 highlighted the potential pitfalls of comparing BMSC and ADSC for cartilage regeneration using *in vitro* culture conditions that were optimised to one cell type, at the expense of the other [71]. Nevertheless, the same authors concluded that while both ADSCs and BMSCs underwent chondrogenic differentiation, it was the latter that produced the greater amount of matrix over a greater range of culture conditions.

In recent years, a number of clinical studies have focused on the chondrogenic potential of ADSCs [71, 74–77]. Jo et al. compared various doses of autologous ADSCs administered via i.a. injection in both phase I and II trials and concluded that better results positively correlated with higher numbers of ADSCs [77]. The highest dose (100 million cells) produced smooth glossy white cartilage that was well integrated with the subchondral bone, comparable to native cartilage and free of calcification at 6 months postinjection. Importantly, in the highest dose, the defect underwent significant reduction in volume paralleled by an increase in cartilage volume in some cases at 6-month follow-up; lower doses of ADSCs did not produce such positive results. In contrast to these findings, a recent clinical trial (NCT01585857) reported that the lowest dose (2 million cells) of autologous ADSCs injected i.a. for knee OA produced the greatest improvement in pain and function tests using the Western Ontario and McMaster Universities Osteoarthritis Index (WOMAC), although this seems to be primarily due to the differences in baseline pain and function seen in the low-dose group [78]. Indeed, little difference is seen at later time points [78].

Further positive results were reported using the autologous stromal vascular fraction (SVF) harvested from the buttocks of 30 patients which were then reinjected intraoperatively to assess the clinical effect on elderly patients with knee OA [72]. Assessment at 2 years showed improvements in motor function, cartilage healing, and reduced pain. After 2 years, the average Lysholm score increased (from 54 to 74), the VAS pain score decreased (from 4.7 to 1.7), and the KOOS increased in all categories at all postoperative time points. The same group followed up the previous study with a level II, prospective comparative study to compare the clinical and radiologic efficacy of ADSCs harvested from the SVF, with fibrin glue and microfractures (MFX) versus MFX alone in 80 patients with knee cartilage defects [73]. The outcomes at 24 months suggest that the addition of ADSCs to MFX protocols could significantly reduce OA pain (reflected in improved MOCART and KOOS scores). The authors reported no significant effects of ADSCs on other matrices measuring daily activity and quality of life.

### 2.5. Peripheral Blood

Cells isolated from the peripheral blood and activated using a combination of the CXCR4 antagonist, AMD3100, and granulocyte colony-stimulating factor have been noted to conform to the criteria defining “MSCs” [79, 80] as laid out by the ISCT. *In vitro* studies using rabbit peripheral blood cells (PBSCs) have shown that not only are these cells substantially more accessible than the corresponding BM-derived cells but that they also possess a greater chondrogenic and adipogenic differentiation potential in *in vitro* assays [79]. In the same *in vitro* tests, BMSCs had a greater osteogenic and proliferative capacity while implantation of both BMSCs and PBSCs produced similar chondrogenic results in an *in vivo* cartilage defect model.

In humans, PBSCs have produced different results when applied to cartilage repair. In a trial comparing 5 weekly injections of HA only or HA plus PBSCs after arthroscopic subchondral drilling for chondral lesions, improvements were noted at 24 months using the IKDC score (*P* = 0.8), using MRI inspection (*P* = 0.013), and using the ICRS score (109-point increase, *P* = 0.022) [81]. It would be interesting to see further studies expanding an essential “cells versus no cells” experiment to include the effects of other stem cells, such as ADSCs or BMSCs. An extension of the above study by the same group repeated the i.a. injections of HA ± PBSCs weekly for 5 weeks and again at 6, 12, and 18 months after arthroscopic subchondral drilling in addition to open-wedge high tibial osteotomy [82]. Assessment of cartilage repair was carried out by histology (ICRS II scoring system) and by second-look arthroscopy indicating that the technique including PBSCs produced cartilage rich in proteoglycans and collagen which closely resembled native cartilage with no adverse effects reported.

### 2.6. Umbilical Cord/Umbilical Cord Blood

Another emerging source of stem cells for tissue regeneration is the umbilical cord; with specific regard to cartilage repair and regeneration, several recent reports have highlighted the potential for these cells in the clinic [83–85]. In a recent case report, autologous umbilical cord blood cells (UBSCs) in a HA hydrogel were implanted in 5 mm diameter and 5 mm deep drilled holes in the lateral femoral condyle. Assessment was at 1 and 5.5 years and showed improvements in VAS (from 46 preop to 8 and 12 at 1 and 5.5 years postop, resp.), IKDC (63.22 preop to 85.02 and 85.5 at 1 and 5 years postop), and WOMAC scores (25 preop to 2 and 4 at 1 and 5 years postop) [86]. Encouragingly at 1 year, second-look arthroscopy revealed no bone formation or bone exposure at the articular surface which was covered with smooth firm hyaline cartilage. MRI at 1 and 5.5 years showed that the defect was filled, that there was smooth integration with the surrounding tissue, and that the repair was maintained over time. In a larger cohort of patients (*n* = 7) assessed at 1, 3, and 7 years postintervention, human allogeneic UBSCs mixed with a HA hydrogel were evaluated for cartilage repair in the femoral condyle [87]. Human UBSCs as with stem cells from other sources have been used allogeneically on the basis of their reputed immunomodulatory properties [84, 88]. Ha et al. used human UBSCs in a hydrogel to examine the repair potential of osteochondral defects in a minipig model and noted no adverse effects, no infection, and no rejection after 12 weeks [83]. Additionally, the UBSC-treated defects, in contrast to the untreated controls, contained GAG-rich cartilage with better integration with the surrounding tissue; the defects which received human UBSCs also did better on the ICRS scoring system.

## 3. Conclusions

The issue of degenerated cartilage will remain a pressing medical need as the world population ages. Tissue engineering represents a valid alternative to current techniques, which can offer temporary or partial relief, but is far from ideal. As illustrated in this review, a wide variety of tissues have been examined for their potential suitability for cartilage regeneration or replacement. Each tissue has different advantages in terms of invasiveness, cell yield, cell proliferation, and chondrogenic potential. Thus, the choice of the best cell source depends on several factors: the intrinsic chondrogenicity, the ease of harvest, and the available cell number. In general terms, it seems that more accessible tissues such as adipose, blood, and umbilical cord tissues have the advantage despite their noncartilage origins. An alternative approach involves the use of allogeneic cells or implants made using allogeneic cells which are subsequently decellularised to overcome this blockade.

This would permit the use of allogeneic cells for cartilage repair, but at the expense of the potentially anti-inflammatory effects of live MSCs [89]. Indeed, downregulation of inflammatory cytokines has been reported in cocultures of ADSCs and osteoarthritic chondrocytes or synoviocytes [90]. Moreover, reductions in reported pain following injection of stromal cell populations from BM [91] and adipose tissue [77, 92] have been reported. Thus, live MSCs may have a central role in pain reduction following a cartilage repair procedure.

Existing techniques, such as autologous or allogeneic chondrocyte implantation, can be optimised by drawing upon fresh insights from basic science and by continuing to experiment with new cell populations. Excitement over basic findings must as always be tempered with caution regarding the safety of treating cells with growth factors and hormones prior to implantation. A balance between guiding cells down the desired lineage path and pushing them over the edge towards malignant transformation is crucial; however, there are few reported instances.

The issue of premature differentiation during 2D *in vitro* expansion is especially salient when discussing explanted articular chondrocytes, which seem to have limited capacity in this regard. Advances in cell culture techniques such as the application of hypoxic growth chambers, as well as 3D perfusion culture utilizing bioreactors that recapitulate not only the 3D in vivo environment but also both hydrostatic and compressive loading [93] found in joints, will be vital to compensate for the low number of progenitors found in articular cartilage. Research into alternate sources of cartilage-forming cells is developing, as illustrated by the range of cell sources covered in this review. Nowadays, autologous mesenchymal cells can already be applied in the clinical settings; in particular, BMSCs or ADSCs can be injected i.a. in case of moderate osteoarthritis with the aim to reduce inflammation and, therefore, pain. In case of focal cartilage lesions, BMSCs can also be used in combination with collagenic membranes to repair the defect. However, the use of MSCs in clinical practice is still limited due to all the issues described above.

Clearly, current methods can generate cartilage *in vivo* with a great promise for future clinical applications. We report on more than 15 case studies or clinical trials in humans with the majority describing positive findings and no adverse effects with follow-up times extending to double-digit figures. This is enormously encouraging, and as we continue to learn more about the nature of progenitor and stem cell populations, we anticipate that improvements in the production of regenerated cartilage will see increased clinical translation and patient benefit.

## Figures and Tables

**(a):** 

Author	Year	Cell source	Model	Experimental study	Adverse effects	Key findings
Shafiee et al.	2011	Cartilage	Mice	Proliferation, tumourigenesis, and multipotency of nasal septum-derived adult cells	None	NCs retained chondrogenic potential until passage 35. Markers suggest chondrogenic ability equal to that of BMSCs
do Amaral et al.	2012	Cartilage	*In vitro*	Proliferation and multipotency of nasal septal cartilage surface zone cells within the context of cartilage repair	NA	Cells in pellet culture resulted in chondrogenesis without TGF-*β* or BMPs. NCs were CD105^+^, CD73^+^, CD44^+^, and CD146^−^
Pelttari et al.	2014	Cartilage	Humans (10), mice, goats	Suitability of adult human neuroectoderm-derived nasal chondrocytes for articular cartilage repair	None	NCs proliferated faster and were more chondrogenic than Acs *in vitro*. *In vivo*, defect filling was observed after 4 months
Jiang et al.	2016	Cartilage	Humans (15), mice	Cartilage repair potential of resident cartilage stem/progenitor cells	None	ACs became CD146^+^ in high-density 2D culture, and their chondrogenic potential is similar to that of BMSCs. *In vivo* results were promising
Embree et al.	2016	Cartilage	Rats, rabbits	Potential of single resident fibrocartilage stem cells (FCSC) to regenerate cartilage, bone, and haematopoietic compartment	None	FCSCs spontaneously produced cartilage anlage *in vivo* which was then remodeled into trabecular bone. Addition of sclerostin maintained the FCSC pool and led to chondrocyte differentiation and cartilage repair *in vivo*
Fellows et al.	2017	Cartilage	*In vitro*	Senescence of healthy versus diseased human knee articular cartilage rather than regenerative potential per se	NA	The number of progenitor cells was greater (2x, *P* < 0.001) in OA tissue than in healthy cartilage. Subpopulation of OA-derived cells had reduced proliferative potential and underwent early senescence *in vitro*. An increase in senescent cells may contribute to the disease phenotype
Pittenger et al.	1999	BM	*In vitro*	Maintenance of multipotency in individual adult BMSCs	NA	Adult stem cells can be induced to differentiate exclusively into adipocytic, chondrocytic, and osteogenic lineages
Wakitani et al.	2004	BM	Humans (2)	Effectiveness of autologous BMSC transplantation for the repair of full-thickness articular cartilage defects in the patellae of 2 individuals	None	Clinical symptoms (pain & walking impediment) were significantly reduced 6 months postop. Benefits remained for 4-5 years. Arthroscopy revealed defects filled with fibrocartilage
Wakitani et al.	2011	BM	Humans (41)	Safety of autologous BMSC implantation for cartilage defects	None	No tumour or infections reported in any patient. Five had total knee replacement due to progression to OA
Wong et al.	2013	BM	Humans (56)	Autologous BMSC i.a. injections with microfracture and tibial osteotomy	None	The experimental group showed significantly better IKDC (*P* = 0.001), Tegner (*P* = 0.021), MOCART (*P* < 0.001), and Lysholm (*P* = 0.016) scores

**(b):** 

Author	Year	Cell source	Model	What was examined	Adverse effects	Key findings
Vangsness et al.	2014	BM	Humans (55)	Safety and effects on OA changes in the knee following intra-articular injection of allogeneic human BMSCs	None	Evidence of meniscus regeneration and improvement in knee pain following treatment with allogeneic human mesenchymal stem cells
Gobbi et al.	2014	BM	Humans (25)	BMAC (BM aspirate concentrate) for the repair of large full-thickness knee cartilage defects	None	Significant improvement in Tegner, Marx, Lysholm, VAS, IKDC subjective, and KOOS scores at the final follow-up compared with their respective preoperative scores (*P* < 0.001); MRI analysis at the final follow-up showed stable implantation and complete filling of the defect in 20 of 25 patients
Vega et al.	2015	BM	Humans (30)	Effects of i.a. injection of allogeneic BMSC versus hyaluronic acid for the treatment of knee OA	None	At 1-year follow-up, cartilage formation in cell-treated defects was significantly improved over control (HA)-treated defects
Nakagawa et al.	2016	BM	Rats	Lubricin expression and chondrogenesis in BMSCs using pellets & hanging-drop cultures *in vitro* and *in vivo*	NS	The treatment group scored significantly higher than the control group when assessed histologically at 8 and 12 weeks
Chen et al.	2016	BM	Rabbits	PTH-treated versus untreated BMSCs embedded in fibrin glue for the repair of induced articular cartilage injury in rabbits	None	The ICRS score significantly increased (*P* < 0.05) in PTH-treated versus non-PTH and untreated groups. Significantly increased levels of type II collagen and aggrecan mRNA and protein in PTH versus non-PTH groups (*P* < 0.05)
Shapiro et al.	2017	BM	Humans (25)	BMAC for the treatment of knee pain from bilateral osteoarthritis	None	Knee pain decreased in all groups, although no significant difference between BMAC and saline groups (*P* > 0.9)
Koga et al.	2008	Synovium	Rabbits	“Local adherent technique” whereby an i.a. injection of synovium stem/progenitor cells adheres to the defect site within 10 minutes	NA	Increased cell attachment correlated with improved cartilage repair at 24 weeks. It was reported that 60% of injected cells adhered at the site
Nakamura et al.	2012	Synovium	Pigs	Adherence of synovium-derived cells to cartilage defects and effects on cartilage	None	The cartilage matrix detected in all treated defects versus none in the control group. Wakitani and ICRS scores were significantly higher in treatment groups (*P* < 0.05). Higher chondrogenic potential in synovial cells versus BM, adipose, muscle, or periosteum-derived cells
Sekiya et al.	2015	Synovium	Humans (10)	“Local adherent technique” using autologous synovium-derived stem/progenitor cells	1 patient had fibrillation of repaired cartilage	Transplantation of synovial cells was deemed effective: Lysholm and MRI-based scores increased over 3 years + follow-up period (both *P* = 0.005)
Mak et al.	2016	Synovium	Mice	Chondrogenic potential of synovium-derived sca-1-positive stem/progenitor cells injected into injured joint	NS	Intra-articular injection of Sca-1^+^ GFP^+^ synovial cells from C57BL6 or MRL/MpJ “super-healer” mice to C57BL6 mice following cartilage injury led to similar levels of cartilage repair. Treatment with cells resulted in cartilage repair that was significantly greater than that of untreated defects
Baboolal et al.	2016	Synovium	Dogs	Role of HA on MSC attachment to cartilage	NS	It was confirmed that HA inhibits MSC-cartilage attachment
Diekman et al.	2010	Adipose	*In vitro*	Differences in chondrogenic potential of ADSC and BMSC in different culture conditions	NA	ADSCs and BMSCs require different *in vitro* culture conditions to achieve optimal chondrogenic outcomes. While both ADSC and BMSC underwent chondrogenic differentiation in all conditions tested, BMSCs produced a more matrix over a wider range of conditions
Koh et al.	2013	Adipose	Humans (18)	Outcome of i.a. injections of autologous ADSCs for the treatment of knee OA	One case of pain and swelling	Significant reduction in WOMAC scores (*P* > 0.001) relative to preop levels. The Lysholm score increased from 40.1 points to 73.4 points (*P* > 0.001), and the mean VAS score decreased over the period of the study from 4.8 to 2.0 (*P* > 0.005)
Jo et al.	2014	Adipose	Humans (18)	Safety and efficacy of i.a. injections of autologous ADSC for knee OA	None	Improvements were seen in the high-dose group (improvement in WOMAC & VAS at 6 months). Significant decreases in cartilage defect size paralleled by an increase in cartilage volume at some defect sites at 6 months
Koh et al.	2015	Adipose	Humans (30)	Injection of ADSCs and arthroscopic lavage for knee OA	Slight knee pain, resolved with medication	The technique appears to be effective in cartilage healing, reducing pain, and improving function
Koh et al.	2016	Adipose	Humans (80)	ADSCs with fibrin glue and microfracture (MFX) versus MFX alone in patients with symptomatic knee cartilage defects	NS	Both treatment groups saw improvement in multiple clinical outcomes; however, the degree of improvement was greater in patients who received ADSC in addition to MFX
Pers et al.	2017	Adipose	Humans (18)	Intra-articular injections of different doses of ADSCs	Unstable angina pectoris reported in 1 patient, 5 minor AEs reported by four patients potentially related to the procedure	All dose groups saw an overall negative trend in WOMAC (pain, stiffness, and function), VAS, and SAS, although these data were significant only in the low-dose group
Saw et al.	2013	Peripheral blood	Humans (50)	Postoperative i.a. injections of hyaluronic acid with and without PBSC	None	A nonsignificant (*P* = 0.8) increase in the IKDC score for the PBSC group at 24 months. A significant (*P* = 0.013) increase in the MRI score in the PBSC group at 18 months
Fu et al.	2014	Peripheral blood	Rabbits	Mobilised rabbit PBSCs versus rabbit BMSCs for *in vivo* chondrogenesis	None	PBSCs showed greater chondrogenic potential than BMSCs *in vitro*, although both cell types performed equally well in *in vivo* assays for cartilage repair
Fu et al.	2014	Peripheral blood	Humans (1)	Injection of autologous activated PBSCs + autologous periosteum flap in a chondral lesion	None	Second-look arthroscopy showed a smooth surface at 8 months postoperation. CT and MRI evaluations showed a significant improvement compared to preoperation
Saw et al.	2015	Peripheral blood	Humans (8)	Autologous PBSCs and HA with concomitant medial open-wedge high tibial osteotomy	None	At 25-month follow-up, arthroscopy and biopsy revealed smooth, well-integrated regenerated tissue rich in type II collagen and proteoglycan, with some type I collagen present
Ha et al.	2015	Umbilical cord	Minipigs	Ability of human UBSC cell lines in HA hydrogel (versus empty defects) to repair osteochondral defects	None	Defects which received cells + HA had more safranin-O-positive staining, more regenerated cartilage, and better integration with the surrounding tissue. The IRCS score was better in cell transplant defects than in empty defects
Li et al.	2016	Umbilical cord	*In vitro*	It was determined whether coculture of human ACs could increase chondrogenic potential of human UBSCs	NA	Indirect coculture increased expression of chondrogenic markers. However, qPCR, WB, and some 2D IHC data contain inconsistencies
Gomez-Leduc et al.	2016	Umbilical cord	Mice	Chondrogenic potential of human UCBSCs seeded on type I/III collagen sponges ± chondrogenic factors	NS	UBSCs cultured *in vitro* with TGF-*β*1 and BMP-2 were implanted in nude mice. Cells exposed to growth factors in an *in vitro* phase produced a cartilaginous matrix rich in type II collagen. No scaffolds progressed to calcification but instead deposited type II collagen-rich ECM
Park et al.	2017	Umbilical cord	Humans (1)	Transplanted human UCBSCs in a 4% HA hydrogel into a rabbit trochlea defect	None	VAS, IKDC, & WOMAC improved. At 1-year follow-up, second-look arthroscopy and biopsy showed smooth safranin-O-positive hyaline-cartilage with excellent peripheral integration. MRI showed defect filling, abundant repair tissue, and good integration with the surrounding tissue
Park et al.	2017	Umbilical cord	Humans (7)	Treatment of a large osteochondral defect by autologous UCBSCs in a HA hydrogel	None	Regenerated tissue was thick, smooth, and glossy white with good integration with the surrounding tissue and resembled hyaline-like cartilage with abundant GAG content. No bone formation or overgrowth was observed
Park et al.	2017	Umbilical cord	Rabbits	Efficacy of human autologous UCBSCs and HA hydrogels for cartilage regeneration	None	Macroscopically, cells + hydrogel produced better cartilage formation than hydrogel only or untreated controls. Regenerated tissue was smooth and type II collagen rich

NA: not applicable; NS: not stated.

## Abbreviations

AC: Articular chondrocyte
ADSC: Adipose tissue-derived stromal/stem cell
BM: Bone marrow
BMAC: Bone marrow aspirate concentrate
BMSC: Bone marrow stromal/stem cell
BMP: Bone morphogenetic protein
CFU: Colony-forming unit
ECM: Extracellular matrix
FCSC: Fibrocartilage stem cell
FGF-2: Fibroblast growth factor 2
GAG: Glycosaminoglycan
HA: Hyaluronic acid
i.a.: Intra-articular
iPSC: Induced pluripotent stem cell
IKDC: International Knee Documentation Committee
ISCT: International Society for Cellular Therapy
KOOS: Knee Injury and Osteoarthritis Outcome Score
MFX: Microfracture
MOCART: Magnetic resonance observation of cartilage repair tissue
MRI: Magnetic resonance imaging
MSC: Mesenchymal/medicinal stem/stromal cell
NC: Nasal chondrocyte
OA: Osteoarthritis
PB: Peripheral blood
PBSC: Peripheral blood stromal/stem cell
RA: Rheumatoid arthritis
SVF: Stromal vascular fraction
TGF-*β*: Transforming growth factor-beta
WOMAC: Western Ontario and McMaster Universities Osteoarthritis Index
VAS: Visual analogue scale.
